# Using Network Extracted Ontologies to Identify Novel Genes with Roles in Appressorium Development in the Rice Blast Fungus *Magnaporthe oryzae*

**DOI:** 10.3390/microorganisms5010003

**Published:** 2017-01-17

**Authors:** Ryan M. Ames

**Affiliations:** Wellcome Trust Centre for Biomedical Modelling and Analysis, Living Systems Institute, University of Exeter, Stocker Road, Exeter, EX4 4QD, UK; r.ames@exeter.ac.uk

**Keywords:** *Magnaporthe oryzae*, rice blast, fungal pathogen, plant pathogen, network, gene expression, RNA-Seq

## Abstract

*Magnaporthe oryzae* is the causal agent of rice blast disease, the most important infection of rice worldwide. Half the world’s population depends on rice for its primary caloric intake and, as such, rice blast poses a serious threat to food security. The stages of *M. oryzae* infection are well defined, with the formation of an appressorium, a cell type that allows penetration of the plant cuticle, particularly well studied. However, many of the key pathways and genes involved in this disease stage are yet to be identified. In this study, I have used network-extracted ontologies (NeXOs), hierarchical structures inferred from RNA-Seq data, to identify pathways involved in appressorium development, which in turn highlights novel genes with potential roles in this process. This study illustrates the use of NeXOs for pathway identification from large-scale genomics data and also identifies novel genes with potential roles in disease. The methods presented here will be useful to study disease processes in other pathogenic species and these data represent predictions of novel targets for intervention in *M. oryzae*.

## 1. Introduction

*Magnaporthe oryzae* is a pathogenic filamentous fungus and the causal agent of the most destructive disease of rice, rice blast [1]. As approximately one-half of the world’s population relies on rice as its primary source of calories [2], *M. oryzae* poses an important threat to global food security. As a result of its agronomic significance and experimental tractability, *M. oryzae* has emerged as a seminal model in the study of host–fungal pathogen interactions [3]. Indeed, in a recent survey, *M. oryzae* was ranked the number 1 fungal pathogen in molecular plant pathology by the international community based on its scientific and economic importance [4].

The early stages of *M. oryzae* infection have been extensively studied. After a three-celled asexual spore (a conidium) lands on a rice plant leaf, one cell of the spore produces a germ tube that extends across the leaf surface. The end of the germ tube swells to produce a melanised dome structure known as an appressorium [5]. Appressorium development is not specific to *M. oryzae* and many pathogenic fungi, such as rusts and powdery mildews, develop these structures for infection. As a precursor to infection, glycerol accumulates in the appressorium causing water to enter via osmosis [6]. In turn, a build up of hydrostatic pressure in the appressorium eventually allows the fungi to burst through the plant cuticle and cell wall and infect the epidermal cells [6].

The pathways involved in appressorium development have also been highly studied. Formation of appressoria is tightly linked to the cell-cycle and requires a DNA replication-dependent checkpoint [7], mitosis [8], migration of a single nucleus from the conidium to the appressorium [9] and infection associated autophagy [8,10]. Targeted gene deletions and replacements have identified several signalling pathways, including cyclic adenosine monophosphate (cyclic-AMP) dependent and mitogen-activated protein (MAP) kinase pathways, important to appressorium development [11,12]. To generate hydrostatic pressure, *M. oryzae* accumulates glycerol in the appressorium by mobilisation of existing glycogen and triacylglycerol lipase activity to release glycerol from stored lipids [13]. Other important pathways for appressorium development include utilisation of acetyl-CoA in gluconeogenesis, fatty acid beta-oxidation and the glyoxylate cycle [14,15]. The involvement of many pathways indicates that appressorium development is a complex process that will involve the highly coordinated expression of many genes.

Several methods have been used to characterise the genes and processes involved in appressorium development. These studies include profiling transcript abundance following appressorium development [16], characterising the response of *M. oryzae* to stresses involved in infection [17,18] and thorough analysis of transcription changes during appressorium development [19]. Although these studies have identified many genes that change expression over the duration of appressorium development, there are likely other genes involved in this process. For example, experimental conditions may not capture the full complexity of the system and may miss the full complement of genes and pathways involved in appressorium development. Alternatively, important genes in appressorium development may have crucial roles in central pathways or participate in multiple pathways (pleiotropy) and thus may not show differential expression in specific experiments. Therefore, to fully understand appressorium development, we need a comprehensive, genome-wide representation of *M. oryzae* that can be interrogated to learn more about the underlying biology.

In the last decade, high-throughput DNA sequencing methods have provided a new method to identify transcriptomes and quantify the level of transcripts. These methods have been applied to yeast [20], *Arabidopsis thaliana* [21], mouse [22] and human [23]. In addition to identifying and quantifying transcriptomes, methods have been developed to identify differential expression of genes [24,25] and infer networks from estimates of expression over multiple samples [26,27,28]. One such method, Clique Extracted Ontology (CliXO), extracts hierarchical networks from gene similarity data such as correlated gene expression and co-expression networks [27]. An ontology consists of entities, in this case groupings or clusters of genes, that are connected by relationships. The structure is hierarchical and multi-scale, so ontologies in biology can represent pathways, complexes and individual genes. Entities can also be related to more than one parent allowing for the representation of pleiotropy in biological networks. So called network-extracted ontologies (NeXOs) have been used to characterise the coordinated expression of a genome, identify the function of uncharacterised genes and identify previously unknown functional links in yeast [29].

In this study, I aggregate several next-generation sequencing studies of *M. oryzae* and estimate genome-wide expression over a variety of conditions including appressorium development. These data are organised into a NeXO where entities are tested for robustness, annotated with biological function and clusters associated with appressorium development are identified. These clusters identify previously reported pathways associated with appressorium development and also identify genes not found in differential expression studies that participate in these pathways. For example, genes associated with the mevalonate pathway, involved in acetyl-CoA processing and genes associated with cell adhesion are identified as potentially important to appressorium development. The results of this study predict genes that might be exploited to disrupt appressorium development and demonstrate the utility of network approaches to analyse next-generation sequencing data that can be applied to other systems.

## 2. Materials and Methods

### 2.1. Genomic and Transcriptomic Data

The genome sequence of *M. oryzae* (70-15 version MG8) was downloaded from the Broad Institute [3]. Annotations for *M. oryzae* were downloaded from the Ensembl Genomes Database [30]. RNA-Seq data were downloaded from the Gene Expression Omnibus (GEO) [31] from three published studies including (i) transcript profiling during appressorium formation (GSE30069) [19]; (ii) transcriptional response to hypoxia in *M. oryzae* (GSE51597) [18]; and (iii) a viability study with inhibited PKC1 (GSE70308) [32]. In total, the expression set contained 46 RNA-Seq samples.

### 2.2. Annotation of M. oryzae Genes

Gene Ontology (GO) terms [33] were assigned to *M. oryzae* sequences using Blast2GO [34] and provided by Darren Soanes [35]. Blast2GO has previously been used to annotate *M. oryzae* genes [19]. Briefly, *M. oryzae* sequences were annotated via sequence similarity using BLAST against the National Center for Biotechnology Information’s (NCBI) non redundant (NR) database with Blast2GO’s default parameters (E-value cutoff 1.0 × 10${}^{-3}$). Next, sequences were searched against the InterPro database, using default parameters, to associate sequences with protein families and include GO term annotations based on protein motifs and domains. Finally, the *M. oryzae* sequences were mapped and annotated with GO terms using default parameters, including an E-value cutoff of 1.0 × 10${}^{-6}$ and applying greater weight to experimentally validated GO term annotations.

### 2.3. Estimating Expression of M. oryzae Genes

An index for the *M. oryzae* genome was built using bowtie2-build and reads from RNA-Seq datasets were aligned to the reference genome using bowtie2 (version 2.2.5) [36]. For both single- and paired-end data, reads were aligned in the “local” alignment mode with the “very-sensitive” preset. Alignment files were post-processed (sorted, converted to binary format) using SAMtools [37]. The number of reads mapping to known genes was estimated using featureCounts (version 1.5.0) in an unstranded manner and using the exon feature in the *M. oryzae* annotation [38]. Expression of genes was estimated by reads per kilobase of transcript per million mapped reads (RPKM) using edgeR (version 3.16.3) with filtering to remove genes with <2 counts per million in >10% of samples [24].

### 2.4. Generating an Ontology of M. oryzae

Estimates of expression for *M. oryzae* genes were correlated across all expression samples for each pair of genes using the absolute Pearson’s Correlation Coefficient (PCC) to calculate a pairwise similarity matrix. In this matrix, genes with highly correlated expression patterns (positive or negative) have a similarity score close to 1 and genes with uncorrelated expression have a value close to 0. CliXO (version 0.3) was used to create an ontology of *M. oryzae* from these correlations in gene expression [27]. CliXO requires a user-defined noise parameter (α) and a parameter to infer missing edges (β). CliXO has been benchmarked on several ’omics data sets for the yeast *Saccharomyces cerevisiae*, including gene expression profile correlations, and it has been shown that α = 0.01 and β = 0.5 produce ontologies that are very similar to that of the Gene Ontology (GO) [27]. In this study, α and β are set to 0.01 and 0.5 respectively.

### 2.5. Estimating the Robustness of the M. oryzae Ontology

The ontology is split into a number of entities with hierarchical relationships between them. As these entities will represent biological pathways and processes, it is important to estimate their robustness with regard to the underlying experimental data. To score the robustness of these gene clusters, a measure of entity quality that considers network support and its robustness to perturbations of input data was taken from Dutkowski et al. [29]. In this measure, network support NS(e) for an entity *e* is defined as the enrichment for co-expression between genes within the cluster, where co-expression is defined as PCC > 0.2. Enrichment (−log(*p*-value)) is estimated using the hypogeometric distribution where the sample size is equal to all possible co-expressed genes in an entity, sample successes are the observed co-expressed genes in that entity, the population size is all possible co-expressed genes in the ontology and population successes are the observed co-expressed genes in the ontology.

To quantify an entity’s robustness to changes in the input data, a bootstrapping approach was employed. Samples were randomly selected with replacement to build an expression set of 46 samples. Correlations for all pairs of genes were calculated and an ontology was inferred as described above. This process was repeated to produce 25 bootstrapped ontologies. Each bootstrapped ontology was then aligned to the original ontology using the method described in Kramer et al. [27]. Alignment was performed using the strict hierarchy model and all nodes were aligned. The bootstrap score B(e) for an entity (e) is then calculated as:

$$B\left(e\right)=\frac{1}{n\sum_{i=1}^{n}S_{i}\left(e\right)}$$

where *n* is the number of bootstrapped ontologies and $S_{i}$ is the alignment score for entity *e* when the original ontology is aligned to the *i*-th bootstrapped ontology. Finally, the robustness score for each entity R(e) is calculated as a geometric mean of the entity’s network support and bootstrap score:

$$R\left(e\right)=\sqrt{NS(e)B(e)}$$

### 2.6. Annotating Entities in the Ontology

Entities within the ontology were functionally annotated by identifying enriched Gene Ontology (GO) terms [33]. Fisher’s exact test was used to identify enriched GO terms in each of these clusters. Briefly, for each entity, each annotated GO term was tested for enrichment where the population size was the number of genes in the *M. oryzae* NeXO and the sample size was the size of the entity. The population successes were the number of genes in the ontology annotated with the specific GO term and the sample successes were the number of genes within the entity annotated with that term. False discovery rate correction was applied to *p*-values using a false discovery rate of 0.05 [39].

### 2.7. Identifying Entities Associated with Appressorium Function

Entities associated with appressorium function were identified as groupings of genes that show enrichment of genes known to be up-regulated between 4 and 8 h or 14 and 16 h during appressorium development [19]. This analysis was limited to clusters whose size was between five and 1000 members. Fisher’s exact test was used to identify enrichment by comparing the proportion of up-regulated genes within each cluster to the proportion of up-regulated genes in the whole ontology. False discovery rate correction was applied to *p*-values using a false discovery rate of 0.05 [39].

## 3. Results

### 3.1. Correlated Expression of M. oryzae Genes

**Figure 1:**
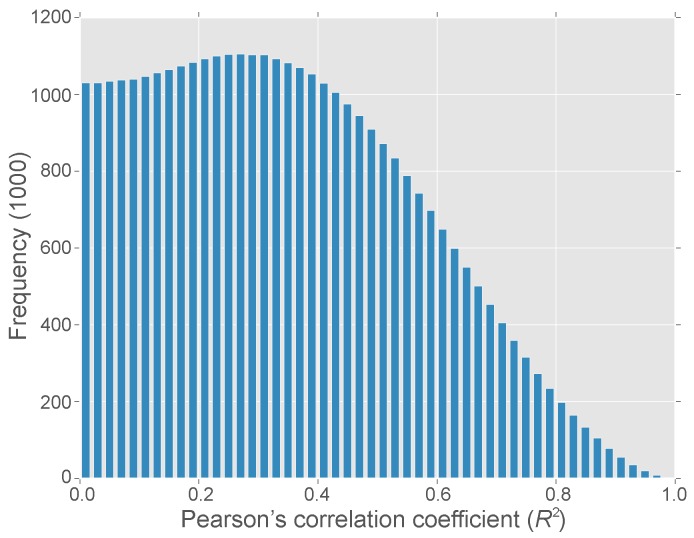
Distribution of correlations for all pairs of *M. oryzae* genes. Pearson’s Correlation Coefficient (R${}^{2}$) are reported as absolute values.

Expression of 8407 *M. oryzae* genes during appressorium development, in response to stress and under gene knockdown conditions from 46 RNA-Seq samples were used to characterise the system. PCC was used to generate a gene similarity matrix. The average correlation between genes is 0.34 ± 0.21 showing that most genes are poorly correlated (Figure 1). Despite low correlations for most genes it is possible to detect genes with highly correlated expression and 2.24% of pairwise comparisons show a PCC > 0.8.

### 3.2. A M. oryzae Network-Extracted Ontology

**Figure 2:**
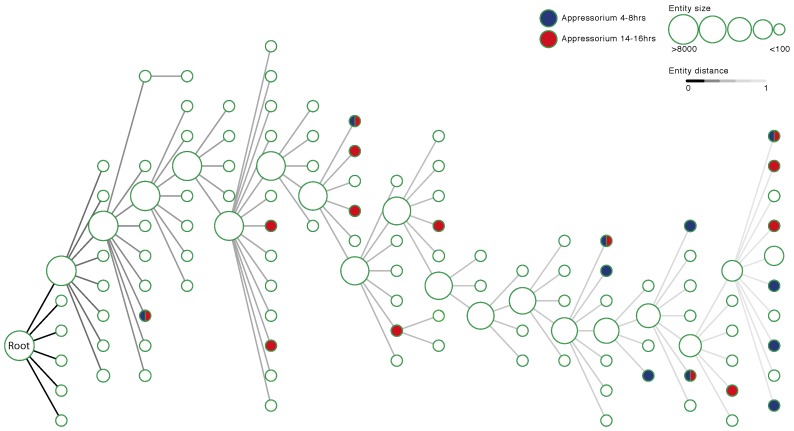
The structure of a *M. oryzae* network-extracted ontology. Nodes represent entities that contain groups of genes and edges represent relationships between entities. Entities are sized by the number of genes they contain and edges are shaded to show a measure of distance between entities. Entities coloured in blue and red depict clusters of genes that are enriched for up-regulated genes at 4–8 h and 14–16 h timepoints during appressorium development respectively. Entities that contain five or more genes are included in the visualisation. The root node that contains the whole system is labelled.

Correlated expression between genes was used to infer a NeXO using the CliXO method [27]. The resulting ontology contained all 8407 genes with expression data organised into a total of 355 entities connected by 4414 entity–entity relationships (Figure 2). The majority of these clusters are small with only 122 containing five or more genes. Likewise, the ontology shows scale-free properties with most clusters having few relationships and a small number of clusters being highly connected. The full structure of the ontology is available in File S1.

### 3.3. Validation of the Ontology Structure

**Figure 3:**
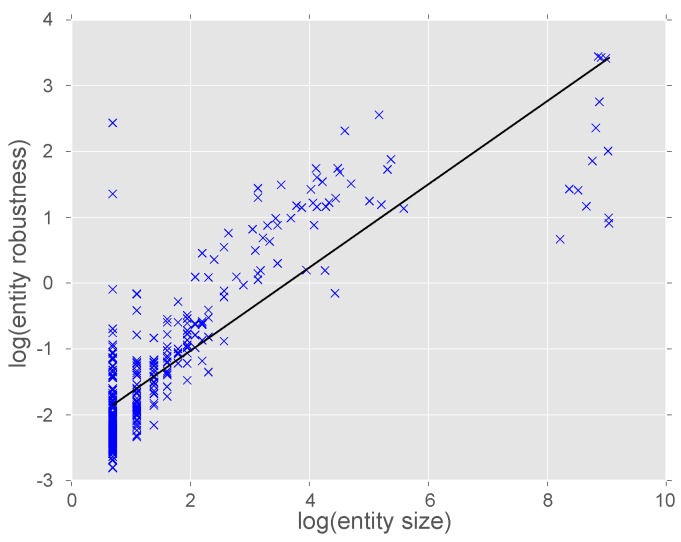
Correlation of entity size and robustness score. Entity robustness is calculated based on network support from co-expression data and from bootstrap analysis. There is a strong positive correlation between entity size and robustness (*R* = 0.84, *p*≪0.0001).

Robustness of the ontology was determined using a bootstrapping approach (see methods). The robustness of each entity was calculated based on network support from the underlying co-expression network and bootstrapping of the input expression data. There is a strong correlation between the size of a cluster and the its robustness (*R* = 0.84, *p*≪0.0001, Figure 3). In general, the larger entities are more robust; for example, excluding those with fewer than 10 members yields an average robustness score of 4.66 compared to 1.10 when all clusters are analysed. There are two clear outliers to this trend (top left Figure 3); CLX0008547 and CLX0008527 both contain only two members but have high robustness scores of 3.87 and 11.39 respectively. This indicates that these small entities consistently contain the same members during the bootstrap analysis.

### 3.4. Identifying Gene and Entity Function

Multiple approaches have been used to annotate *M. oryzae* genes with GO terms. A previous study, that used similarity searches and literature curation to assign GO terms, annotates 5867 (69.78%) of the 8407 genes in the *M. oryzae* ontology with on average 2.09 ± 0.99 terms [40]. In this data set, no genes in this data set are annotated with any of the 28 appressorium related terms in the GO. *M. oryzae* genes can also be assigned with GO terms using Blast2GO [34], which has been used in previous *M. oryzae* studies [19]. An updated version of the Blast2GO annotations has been provided by Darren Soanes [35]. In these data, 6162 (73.29%) of the 8407 genes used in this study were annotated with GO terms and, on average, these genes were annotated with 5.22 ± 4.04 terms. These annotations are also based on high sequence similarity during the annotation process with an e-value cutoff of 1.0 × 10${}^{-3}$ and average sequence similarity of 77.34% ± 14.99, suggesting that these annotations are likely to have a high level of accuracy. In order to get the greatest coverage of annotation and utilise the largest number of GO terms the Blast2GO annotations were used in this study.

GO terms enriched in gene clusters from the *M. oryzae* ontology were identified (Table S1). Only 40 (11.26%) clusters of 355 show significant enrichment of GO terms with each cluster enriched for on average 6.57 ± 9.74 terms. The sparsity and high variability in cluster GO term enrichment may be a consequence of the clusters being specific to appressorium formation. The ontology is mostly constructed from expression during appressorium development and only a single gene in this study is annotated with a GO term relating to appressorium development (MGG_03860 — GO:0075039) suggesting that the GO annotations from Blast2GO may not fully represent this process.

Despite the difficulty in attributing function to entities in the *M. oryzae* ontology, several core and pathogenicity-related functions are represented. For example, CLX0008671, CLX0008467 and CLX0008611 are associated with core pathways and processes such as translation (including; GO:0070993, GO:0006413, GO:0008135), ion transmembrane transport (including; GO:0015992, GO:0098655, GO:0015078) and cellular structure organisation (including; GO:0007010, GO:0030865, GO;0000226) respectively. Entities with function related to pathogenesis include CLX0008737 that is annotated with GO terms related to interaction with another organism via secreted substances (GO:0052047) and CLX0008720 and CLX0008606 that are annotated with polyketide metabolism (GO:0030638), which consists of genes known to be important for pathogenicity [41]. As annotations based on the GO do not represent appressorium development, a different method is necessary to identify pathways and genes involved in this process.

### 3.5. Identifying Entities Enriched for Appressorium Development Genes

Entities related to appressorium development were defined as those clusters that are enriched for genes known to be up-regulated during the development of the infection structure [19]. Eleven entities in the *M. oryzae* ontology are enriched for genes up-regulated during 4–8 h of appressorium development (Figure 2 and Table 1). These clusters range in size from almost 200 genes (CLX0008738) to only 18 genes (CLX0008701). Full lists of genes in these clusters can be found in Table S2. These clusters of genes are hypothesised to function in appressorium development as they are enriched for up-regulated genes during this process. Furthermore, the non-regulated genes in these clusters may be important for appressorium development and function in relevant pathways but are not detectable by differential expression analysis.

**Figure 4:**
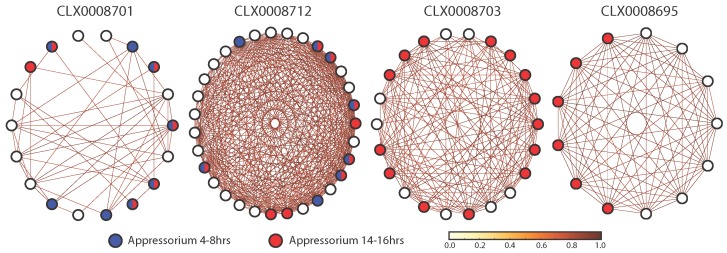
Examples of entities enriched for up-regulated *M. oryzae* genes during appressorium development. CLX0008701 and CLX0008712 are enriched for genes during both 4–8 h and 14–16 h of appressorium development. Entities CLX0008712 and CLX0008695 are enriched for genes up-regulated during 14–16 h of appressorium development. Nodes represent genes and edges between nodes represent correlated expression of genes across 46 RNA-Seq samples. Edges are coloured by Pearson’s Correlation Coefficient (*R${}^{2}$*) and only shown if *R${}^{2}$*> 0.9. Genes up-regulated during 4–8 h and 14–16 h of appressorium development are coloured blue and red respectively.

CLX0008701 is enriched for up-regulated genes during appressorium development (Table 1 and Table 2). This group of genes contains 18 genes of which 8 and 6 are up-regulated during 4–8 h and 14–16 h of appressorium development respectively. A single gene involved in the mevalonate pathway, 3-hydroxy-3-methylglutaryl-coenzyme A reductase (HMG-CoA, MGG_08975), is present in this entity and up-regulated during appressorium development [19,42]. Interestingly, PMD1 (MGG_08970), a leptomycin resistance B protein, flanks MGG_08975 in the *M. oryzae* genome but is not up-regulated during appressorium development. The results suggest that these genes may be functionally linked as they co-occur in an entity with high co-expression over multiple timepoints and conditions (Figure 4).

During 14–16 h of appressorium development, 14 entities are enriched for up-regulated genes ranging in size from 99 to five genes (Figure 2 and Table 2). Full lists of the genes in these clusters can be found in Table S3. One of these, CLX0008703, contains 22 genes with 14 showing up-regulation during appressorium development (Figure 4). The cluster contains genes related to sugar transport (MGG_05889, MGG_08776), sugar processing (MGG_02103, MGG_08569) and oxidoreductase activity (MGG_01387, MGG_10239, MGG_09212). This grouping of genes also contains genes relating to the processing of acyl-CoA (MGG_16316, MGG_03774). The enrichment of genes up-regulated in the later stages of appressorium development suggests that sugar transport, sugar utilisation and acyl-CoA processing may be important in the disease process.

CLX0008695 has 13 members with six showing significant up-regulation during 14–16 h of appressorium development (Figure 4). The up-regulated genes include a 3-oxo-5-alpha steroid 4-dehydrogenase 2 (MGG_03905), a high affinity glucose transporter (MGG_05946), a novel fasciclin (MGG_02884), a histone-lysine methyltransferase (MGG_06852) and two hypothetical proteins (Table S3). Other genes in this entity include a gene associated with flavin adenine dinucleotide (FAD) binding (MGG_16834), some potential membrane proteins (MGG_03584 and MGG_06854) and a FAD dependent oxidoreductase superfamily gene (MGG_01386). Overall, this cluster seems to represent membrane bound proteins that have function related to transport and cell adhesion.

There are five entities enriched for up-regulated genes in both the 4–8 h and 14–16 h timepoints during appressorium development (Table 1 and Table 2). This suggests that the pathways and processes described by these clusters are important for the duration of appressorium development. CLX0008712 is enriched for up-regulated genes throughout the development of appressoria. The co-expression network that defines this entity is almost entirely connected with the genes showing highly correlated expression profiles (Figure 4). This result strongly suggests that this grouping of genes are functionally related. CLX0008712 contains a mix of genes including membrane proteins and transporters (MGG_05062, MGG_15203, MGG_17002), genes linked to mycelium development (MGG_02814, MGG_04740) and a gene (MGG_09372) whose protein contains a fascilin domain and may be involved in cell adhesion. Interestingly, CLX0008712 contains a member of the mevalonate pathway (MGG_09750), which is also represented by CLX0008701, suggesting that these entities may describe overlapping functions or pathways.

## 4. Discussion

Ontologies extracted from network data have been used to identify the function of uncharacterised genes [29]. These NeXOs have been proposed as useful tools to predict a range of cellular phenotypes and answer important biological questions [43]. In this study, RNA-Seq data were aggregated for the rice blast fungus *M. oryzae* and expression profiles were correlated for all pairs of genes. These similarity measures were used to infer a NeXO that aims to represent the process of appressorium development in *M. oryzae*. The ontology contains entities, clusters of genes, linked by relationships (Figure 2). These clusters were annotated with functions using the GO and larger clusters; particularly those with >10 members were shown to be robust to bootstrap analysis (Figure 3). Overlaying information on genes significantly up-regulated during appressorium development was used to identify pathways and genes potentially associated with this process and important for pathogenicity.

CLX0008701 is enriched for up-regulated genes during 4–8 h and 14–16 h of appressorium development and several members of the entity have previously been associated with pathogenicity. Both PMD1 (MGG_08970), a leptomycin B resistence protein, and a HMG-CoA reductase (MGG_08975) are co-located on chromosome 2 and present in CLX0008701. Interestingly, the gene cluster containing these genes, a regulatory enzyme (MGG_08974) and a polyketide synthase gene (MGG_08969), have previously been implicated to play an important role in the pathogenicity of *M. oryzae* [41,44,45]. Additionally, this cluster also contains MGG_00503, a diacyglycerol O-acyltransferase, which is implicated in the glycerolipid pathway in the Kyoto Encyclopedia of Genes and Genomes (KEGG) [46]. The utilisation of glycerol from stored lipids is necessary for the development of turgor in the appressorium [13,14,15]. These results suggest that clusters within the ontology are grouping together genes and processes involved in appressorium development and pathogenicity.

Other members of CLX008701 have also been linked with disease and pathways involved in virulence. HMG-CoA reductase, which is significantly up-regulated during appressorium development is a key enzyme in the mevalonate pathway. The mevalonate pathway converts acetyl-CoA to isopentenyl pyrophosphate (IPP) and dimethylallyl pyrophosphate (DMAPP) [47]. Two species of phytopathogenic fungi, *Botrytis cinerea* and *Cercospora pini-densiflorae*, have been shown to produce abscisic acid (ABA) via the mevalonate pathway [48]. *M. oryzae* contains an orthologous group of genes to the ABA gene cluster in *B. cinerea* and these genes have been shown to contribute to virulence [49,50]. The results suggest that HMG-CoA reductase and more generally the mevalonate pathway may be important for pathogenicity in *M. oryzae* and a range of phytopathogenic fungi. Indeed, in *F graminearum* the HMG-CoA reductase HMR1, has been shown to be an essential gene and has been linked to pathogenesis via the production of secondary metabolities from the mevalonate pathway [42].

One key advantage of the hierarchical structure of ontologies is that they are capable of capturing function at multiple-scales [51]. Indeed, the *M. oryzae* ontology represents the mevalonate pathway at multiple scales. The mevalonate pathway consists of two main parts; the first converts acetyl-CoA to mevalonate and includes the enzyme HMG-CoA reductase. The second part of the pathway converts mevalonate to IPP and DMAPP through several intermediaries and the action of multiple enzymes. CLX0008701 represents the first part of the pathway as the entity contains HMG-CoA reductase. CLX0008712 represents, in part, the second stage of the mevalonate pathway and contains MGG_09750, which encodes a diphosphomevalonate decarboxylase that is involved in converting mevalonate to IPP. Furthermore, CLX0008712 is also enriched for genes up-regulated during appressorium development (Table 1), adding further evidence that the mevalonate pathway is involved with appressorium development and pathogenicity.

CLX0008703 is enriched for up-regulated genes during the later stage of appressorium development (Figure 4). The entity appears to be involved in sugar transport (MGG_05889, MGG_08776), sugar metabolism (MGG_02103, MGG_08569) and contains oxidoreductases (MGG_01387, MGG_10239, MGG_09212) as well as other reductases (MGG_08568, MGG_07576). This result is in line with the hypothesis that *M. oryzae* uses the appressorium to prepare for infection of epidermal cells and to utilise host sugars for nutrition [19]. Interestingly, this cluster contains MGG_16316, a hypothetical protein annotated with acyl-CoA dehydrogenase activity. The utilisation of acetyl-CoA has been shown to be important for appressorium formation through lipid metabolism [15] and the glyoxylate cycle [14]. The glyoxylate cycle has been shown to be important for pathogenesis in *Mycobacterium tuberculosis* [52] and *Candida albicans* [53] indicating similarities in pathogenesis across these species.

Previous studies have shown that membrane proteins, transmembrane transporters and secreted proteins involved in cell adhesion are important for a variety of pathogenic processes in *M. oryzae* [19,54]. CLX0008965 contains several genes associated with the cell membrane that may play a role in transport and cell adhesion. One such gene, FLP1 (MGG_02884), encodes a novel fasciclin-like protein. Indeed, proteins containing fasciclin domains have been associated with cell adhesion in animals [55,56]. FLP1 deletion in *M. oryzae* results in a reduction of conidial adhesion and a reduction in appressorium turgor suggesting a role in adhesion, appressorium development and pathogenicity [54]. FLP1 is up-regulated during 14–16 h of appressorium development [19] and is present in two entities (CLX0008695 and CLX0008726) that are enriched for up-regulated genes in appressorium formation. Interestingly, other genes annotated as fasciclin domain proteins can be found in other enriched entities, for example MGG_09372 is present in CLX0008712. These results highlight the ability of NeXOs to identify pathways and processes involved in *M. oryzae* pathogenicity, and these pathways contain genes that may encode novel proteins involved in appressorium development.

This study has demonstrated the utility of novel network analyses of genomic data for the study of infectious diseases of plants. Network approaches have previously been applied to study *M. oryzae*. He et al. [57] have inferred protein–protein interactions to build a physical interaction network of *M. oyzae*. The authors find that pathogenicity genes tend to be highly connected in the network and interestingly often cluster with genes associated with ion and protein transport [57]. There are similarities with the work presented here, which identifies clusters of genes that are enriched for pathogenicity genes can be associated with transmembrane transport, such as CLX0008965, further stressing the importance of transporter activity in *M. oryzae* pathogenesis. The techniques used here, and network approaches in general, will be transferable to study a range of diseases in plants and animals. This work demonstrates that NeXOs built from network data can be interrogated to generate hypotheses for important biological questions and, therefore, may represent an important advancement in the analysis of large-scale ’omics data [43,51].

It is important to note that the underlying data used to infer NeXOs is crucial to the types of questions that can be answered. Previous studies have used NeXOs to describe broad functional categories for the yeast *S. cerevisiae* and infer the function of uncharacterised genes by predicting GO term annotation [29]. This was made possible by utilising integrated data from protein–protein interactions, gene co-expression and genetic interactions, which represent varied areas of biological function [28]. The ontology constructed here predominantly represents appressorium development in *M. oryzae* as the RNA-Seq data underlying the ontology is dominated by data collected during appressorium formation [19]. Therefore, the NeXO constructed in this study is primarily useful for the identification of pathways and genes with potential roles in appressorium development rather than functional annotation of all uncharacterised *M. oryzae* genes. Future studies will need to ensure that research questions are suitable for the data available.

Analysis of the *M. oryzae* ontology has revealed several pathways involved in appressorium development and pathogenicity. Although many of these pathways have previously been associated with pathogenicity through knockout experiments and transcriptomic analysis, this study has identified novel pathways and genes implicated in disease. For example, genes whose proteins are thought to contain fasciclin domains and might be involved in cell adhesion [54] are present in multiple entities enriched for genes up-regulated during appressorium development. Although these genes are not up-regulated during this process, they are strongly co-expressed with appressorium related genes and therefore act as novel candidates of genes involved in pathogenicity. Furthermore, the use of a NeXO captures the relationships between these pathways and genes. For example, the mevalonate pathway is represented at multiple scales with the two parts of the pathway split into two clusters, which suggests some functional partitioning of this pathway and demonstrates associations with other genes involved in appressorium formation. Ultimately, this study has identified novel genes related to appressorium development that may represent new candidates for anti-fungal targets and intervention in rice blast disease.

## Figures and Tables

**Table 1:** Entities enriched for genes up-regulated at 4–8 h during appressorium development. Entities highlighted in bold are enriched for appressorium genes at both 4–8 h and 14–16 h timepoints.

Entity ID	Up-Regulated Genes	Entity Size	p−raw	p−corr
CLX0008732	16	88	0.007	0.016
**CLX0008701**	8	18	8.776 × 10${}^{-5}$	3.510 × 10${}^{-3}$
CLX0008738	30	183	0.001	0.004
**CLX0008733**	29	91	1.268 × 10${}^{-9}$	2.537 × 10${}^{-8}$
CLX0008723	15	61	2.959 × 10${}^{-3}$	9.864 × 10${}^{-3}$
CLX0008717	16	44	7.793 × 10${}^{-7}$	5.195 × 10${}^{-6}$
CLX0008704	6	23	0.014	0.029
CLX0008709	11	25	4.678 × 10${}^{-6}$	2.339 × 10${}^{-5}$
**CLX0008712**	8	31	0.005	0.013
**CLX0008718**	18	48	9.465 × 10${}^{-8}$	9.465 × 10${}^{-7}$
**CLX0008721**	11	58	0.018	0.032

**Table 2:** Entities enriched for genes up-regulated at 14–16 h during appressorium maturation. Entities highlighted in bold are enriched for appressorium genes at both 4–8 h and 14–16 h timepoints.

Entity ID	Up-Regulated Genes	Entity Size	p−raw	p−corr
CLX0008666	5	7	3.535 × 10${}^{-3}$	0.001
CLX0008638	3	5	0.012	0.030
CLX0008664	5	7	3.535 × 10${}^{-3}$	0.001
CLX0008726	34	69	9.521 × 10${}^{-15}$	2.951 × 10${}^{-13}$
**CLX0008701**	6	18	0.012	0.030
CLX0008703	14	22	1.022 × 10${}^{-8}$	1.584 × 10${}^{-7}$
CLX0008734	20	99	0.011	0.030
CLX0008695	6	13	0.001	0.008
CLX0008731	19	85	0.005	0.021
**CLX0008733**	25	91	4.043 × 10${}^{-5}$	3.133 × 10${}^{-3}$
CLX0008722	14	59	0.007	0.022
**CLX0008712**	9	31	0.006	0.022
**CLX0008718**	11	48	0.021	0.048
**CLX0008721**	23	58	4.601 × 10${}^{-8}$	4.754 × 10${}^{-7}$

## Supplementary Materials

The following are available online at http://www.mdpi.com/2076-2607/5/1/2/s1, File S1: The *M.oryzae* ontology structure; columns 1 and 2 represent entities and genes connected by relationships, column 3 defines the relationship and column 4 defines the distance between entities, Table S1: Gene Ontology terms enriched in entities of the *M. oryzae* ontology, Table S2: Descriptions of entities enriched for up-regulated genes during 4–8 h of appressorium development, Table S3: Descriptions of entities enriched for up-regulated genes during 14–16 h of appressorium development.
